# Mitogenome-Based Phylogeny with Divergence Time Estimates Revealed the Presence of Cryptic Species within Heptageniidae (Insecta, Ephemeroptera)

**DOI:** 10.3390/insects15100745

**Published:** 2024-09-26

**Authors:** Zhi-Qiang Guo, Chen-Yang Shen, Hong-Yi Cheng, Yu-Xin Chen, Hui-Yuan Wu, Kenneth B. Storey, Dan-Na Yu, Jia-Yong Zhang

**Affiliations:** 1 College of Life Sciences, Zhejiang Normal University, Jinhua 321004, China; 2 Department of Biology, Carleton University, Ottawa, ON K1S 5B6, Canada; 3 Key Lab of Wildlife Biotechnology, Conservation and Utilization of Zhejiang Province, Zhejiang Normal University, Jinhua 321004, China

**Keywords:** mitogenomes, Heptageniidae, phylogenetic relationship, divergence time, cryptic species

## Abstract

**Simple Summary:**

Heptageniidae is the third most species-rich family within Ephemeroptera (mayflies). Although multiple studies have been conducted, the monophyly, phylogenetic relationships, and the divergence time of its subfamilies have always been controversial. The current study sequenced 17 new mitogenomes to reconstruct the phylogenetic relationships and calculate the divergence time within Heptageniidae. Therefore, based on comparing the composition of three mitogenomes bias, phylogenetic relationships, and divergence time of *Epeorus montanus* Brodsky, 1930, we suggest that cryptic species exist in *E. montanus*.

**Abstract:**

Heptageniidae are known for their flat heads and bodies and are divided into three subfamilies. Despite the extensive diversity within this group and considerable efforts made to understand their evolutionary history, the internal classifications and origin time of Heptageniidae remains controversial. In this study, we newly sequenced 17 complete mitogenomes of Heptageniidae to reconstruct their phylogenetic positions within this family. Because of the ambiguous time of origin, our study also estimated the divergence time within Heptageniidae based on five fossil calibration points. The results of BI and ML trees all highly supported the monophyly of Heptageniidae and three subfamilies. The phylogenetic relationship of Rhithrogeninae + (Ecdyonurinae + Heptageniinae) was also recovered. The divergence time showed that Heptageniidae originated from 164.38 Mya (95% HPD, 150.23–181.53 Mya) in the mid-Jurassic, and Rhithrogeninae originated from 95.54 Mya (95% HPD, 73.86–120.19 Mya) in the mid-Cretaceous. Ecdyonurinae and Heptageniinae began to diverge at 90.08 Mya (95% HPD, 68.81–113.16 Mya) in the middle Cretaceous. After morphological identification, analysis of the mitogenome’s composition, genetic distance calculation, phylogenetic analysis, and divergence time calculation, we suggest that two different populations of *Epeorus montanus* collected from Aksu, Xinjiang Uygur Autonomous Region (40°16′ N, 80°26′ E) and Xinyuan, Xinjiang Uygur Autonomous Region (43°20′ N, 83°43′ E) in China are cryptic species of *E. montanus*, but further detailed information on their morphological characteristics is needed to fully identify them.

## 1. Introduction

Mayflies are an ancient group of pterygote insects that are found globally, inhabiting freshwater ecosystems everywhere except Antarctica [1,2]. Heptageniidae (Insecta: Ephemeroptera) is the third most species-rich family within Ephemeroptera and encompasses three subfamilies (Heptageniinae, Rhithrogeninae, Ecdyonurinae), with 37 genera and more than 606 species recorded [3,4,5,6]. Initial classification of the Heptageniidae was primarily derived from characteristics of the adult male, with a particular emphasis on the length ratios among the fore tarsal segments from studies in the early to mid-19th century [7]. Since then, some new characters have been used for classification, such as the submentum in larvae being vestigial, the labium being extensively modified, and the first tarsal segment is clearly connected to the tibia on all legs [4,8,9]. Lacking sufficient molecular data, the classification of Heptageniidae remains controversial, and the phylogenetic relationships among the three subfamilies has remained unstable. At the family level, Ball et al. failed to recover the monophyly of Heptageniidae on the basis of the *COI* gene [10]. Similarly, on the basis of a dataset of *12S rRNA*, *16S rRNA*, *18S rRNA*, *28S rRNA* and *H3* genes, Odgen did not recover the monophyly of Heptageniidae [11], nor did the research of Sun et al. [12]. However, more studies supported the monophyly of Heptageniidae [13,14,15,16,17,18,19,20,21,22,23,24]. For its sister clade, Cai et al. [13] recovered the relationship of Heptageniidae + (Isonychiidae + (Siphlonuridae + Ameletidae)) from 13 protein-coding genes (PCGs) of the mitogenome. Yu et al. [25] supported a closer relationship of Heptageniidae with Isonychiidae that was consistent with Odgen et al. [26] and Wang et al. [27]. However, some researchers suggested that Heptageniidae was the sister clade to the branch containing Baetidae, Neoephemeridae, Leptophlebiidae, Ephemerellidae, Ephemeridae, Vietnamellidae, Potamanthidae, Caenidae, Polymitarcyidae, and Teloganodidae rather than Isonychiidae, Siphlonuridae, or Ameletidae [15,18,19,20,22,23,24]. At the subfamily level, many studies did not recover the monophyly of the three subfamilies; for example, Zurwerra et al. [28] did not successfully recover the monophyly of Ecdyonurinae when reconstructing the phylogenetic relationships within European Heptageniidae on the basis of a combination of biochemical and morphological data. Ma et al. [29] did not recover the monophyly of Ecdyonurinae from 13 PCGs. Therefore, García-Giron et al. [30] also did not recover the monophyly of the three subfamilies. Debate over the subfamily relationships of Heptageniidae also exist. Webb et al. [31] supported the phylogenetic relationship of Rhithrogeninae + (Ecdyonurinae + Heptageniinae) on the basis of the *COI* gene, and the results were similar to those of Wang et al. [32]. On the contrary, Xu et al. [21] suggested the phylogenetic relationship of Heptageniinae + (Ecdyonurinae + Rhithrogeninae) using the dataset of 13 PCGs, which was consistent with the results of Tong et al. [18].

Ephemeroptera are often thought to have originated in the Late Carboniferous or Early Permian [33,34]. The Mesozoic mayfly fossils are relatively more abundant than other periods to date, but few can be linked to extant mayflies [35]. Several studies have already been provided but the divergence time estimations of these taxa among Ephemeroptera is still inadequate. For instance, Misof et al. [36] estimated the origin time within major orders of Insecta, and the results showed that the Ephemeroptera originated at approximately 239 million years ago (Mya), whereas García-Giron et al. [30] supported the idea that mayflies began to diverge at around 178.82 Mya, and Kohli et al. [37] suggested that mayflies appeared at about 267.14 Mya. Regarding the issue of the origin time of Heptageniidae, Tong et al. [19] estimated the divergence time within Ephemeroptera on the basis of four fossil calibration points and showed that the Heptageniidae diverged 173.64 Mya in the mid-Jurassic, and García-Giron et al. [30] also suggested that Heptageniidae originated 134.57 Mya in the Late Jurassic. At the subfamily level, Vuataz et al. [38] used the formation time of the Aegean Trench to estimate the Alpine Rhithrogeninae and suggested an origin at 21.6 Mya.

William Derham was first to advance the concept of cryptic species in the genus *Phylloscopus* (Passeriformes, Phylloscopidae) [39], and Struck et al. [40] redefined it (two or more distinct species being erroneously classified under one species name), which gained widespread acceptance among most researchers. The issue of cryptic species within Ephemeroptera is a hot topic that has emerged in recent years, and most researchers have focused on Baetidae [18,19,38,41,42,43]. Stauffer-Olsen [41] detected potential cryptic species within *Baetis* by comparing the *COI* gene within *Baetis* (Baetidae). Both Bisconti et al. [42] and Williams et al. [44] found distinct and deeply divergent species within the *Baetis rhodani* (Baetidae) group. Rutschmann et al. [43] suggested the presence of cryptic species in *Baetis* and *Cloeon* (Baetidae) in light of molecular data (*COI*) and morphological characteristics. Later, Tong et al. [19,20] found cryptic species within *Vietnamella sinensis* (Vietnamellidae) and *Siphluriscus chinensis* (Siphlonuridae) by comparing their complete mitogenome characteristics, phylogenetic relationships, genetic distance, etc. In Heptageniidae, Lalueza-Fox et al. [45] suggested the presence of cryptic species within *Rhithrogena* by studying the *COI* and *PEPCK* genes.

Mitochondria are widely found in eukaryotes (one of their key characteristics), and in addition to providing more than 95% of the ATP energy needed for physiological activities, these organelles also play crucial roles in insects, such as dealing with oxidative stress, diapause, diseases, and immunity [46,47]. Because of maternal inheritance and the higher evolutionary rate, modern studies of mitogenomes always employ mitochondria in order to explore the phylogenetic relationships and cryptic species among Insecta [18,19,41,48,49,50,51,52,53]. With a size ranging across 14–20 kb and containing 37 genes including 13 PCGs, 22 transfer RNAs (tRNAs), two ribosomal RNA genes (rRNAs), and a non-coding region (CR, also known as an AT-rich region), the arrangement order of the 37 genes in the mitogenome of Ephemeroptera is always considered to be consistent with that of *Drosophila yakuba* [47,54,55,56]. However, there is a change in gene order or gene content in some cases, and this seems to always occur in genes near the CR [57]. In Ephemeroptera, gene rearrangements are not uncommon, as the rearrangement order of *trnI-CR-trnC-trnQ-trnY-trnM-ND2-trnW* was found in *Alainites yixiani*, whereas *Siphluriscus chinensis* was characterized by *trnS1-trnK-trnE* [58], and two gene clusters of *trnI*-*CR*-*trnQ*-*trnM* and *trnI*-*trnI*-*trnI*-*CR*-*trnQ*-*trnM* appeared in Ephemerellidae [22]. Two new gene clusters (*trnI-trnM-trnQ-trnM* and *trnI-trnM-trnQ-NCR-ND2*) were found in the Heptageniidae mitogenomes released by the NCBI (https://www.ncbi.nlm.nih.gov, accessed on 10 April 2024), except for *Paegniodes cupulatus* and *Rhithrogena germanica*, which showed the primitive gene cluster of *trnI-trnQ-trnM* [17]. Diverse gene rearrangement structures are considered to carry significant genetic information; for example, Xu et al. [21] demonstrated that the rearrangements within Heptageniidae bore a certain relationship with their phylogenetic relationships.

During the process of using the Basic Local Alignment Search Tool (BLAST) on NCBI [59], we found there are two sequences of the *COI* gene and one complete mitogenome of *E. montanus* released by the NCBI. And we also found that two samples collected in this study were matched with one *COI* gene each but failed to match with the other two. After morphological identification, calculating the genetic distance, phylogenetic analysis, and divergence time calculation, we suggested the presence of the cryptic species in *E. montanus*. To investigate the phylogenetic position of the Heptageniidae within Ephemeroptera and its internal phylogenetic relationships, we also newly sequenced 17 complete mitogenomes (including two mitogenomes of *E. montanus*). In light of the data above, the main objectives in this study were to: (1) discuss the phylogenetic relationship of Heptageniidae within Ephemeroptera, (2) assess the origin time of Heptageniidae on the basis of five fossil calibration points, and (3) discuss the cryptic species of *E. montanus*.

## 2. Materials and Methods

### 2.1. Taxon Sampling and Species Identification

In order to expand the sample size and sampling range, in this study, sampling was conducted in 14 different locations across five provinces during 2022 to 2023 (Figure S1). In total, 17 samples of 15 species were stored in Zhang’s lab, College of Life Sciences, Zhejiang Normal University, Zhejiang, China. All samples were preserved in 100% ethanol and stored at −20 °C. Detailed information about these samples is shown in Table 1. Via analyses of the morphological characteristics of the leg, head, antenna, maxilla, labium, hypopharynx, mandible, labrum and cercus, we identified the species under an Olympus SZX16 stereomicroscope (Olympus Corporation, Tokyo, Japan). Then we extracted the total genomic DNA by using an Ezup Column Animal Genomic DNA Purification Kit (Sangon Biotech Company, Shanghai, China) following the manufacturer’s standard operating procedures.

### 2.2. Mitogenome Sequencing and Assembling

After extracting the total genomic DNA, we used the universal primers LCO1490: 5′-GGTCAACAAATCATAAAGATATTGG-3′ and HCO2198: 5′-TAAACTTCAGGGTGACCAAAAAATCA-3′ to amplify the *COI* gene [60]. Species were further identified by comparing the *COI* gene’s sequence through BLAST from the NCBI. In this study, the mitogenomes were obtained by combining the Sanger sequencing method and high-throughput sequencing. To better understand the mitogenome, one mitogenome (*Epeorus aculeatus*) was obtained via the Sanger sequencing method, and 13 pairs of universal primers referenced from Zhang et al. [61] were used to conduct polymerase chain reaction (PCR) tests. All PCR products were sequenced by Sangon Biotech Company (Shanghai, China). Individual gene fragments were assembled using SeqMan v.5.01 in the DNAstar software package [62]. To save costs and time, the remaining 16 mitogenomes were sequenced via high-throughput sequencing by BerryGenomics (Beijing, China). After obtaining raw sequences, SOAPnuke and Berry fastqc_pe were used for primary quality control. For the reads that had low-quality bases, a high ‘N’ base content, and adapter sequence, when the base content of ‘N’ was high in single-end sequencing reads, we removed such pairs of reads. To enhance the credibility of the final mitogenomes, NOVOPlasty v.4.2 [63], GetOrganelle v.1.7.1 [64], MitoZ [65], and MitoFinder v.1.4.7 [66] were used to assemble the mitogenomes on the basis of the clean data. After we obtained the same mitochondrial genomes by at least four methods, we judged that the mitogenomes were correct.

### 2.3. Mitogenome Annotation and Structural Analysis

The positions of tRNAs were identified by combining MITOS2 service in Galaxy (usegalaxy.eu, accessed on 10 April 2024) [67] with tRNAScan-SE 2.0 (lowelab.ucsc.edu/tRNAscan-SE, accessed on 11 April 2024) [68]. By using ClustalW in MEGA v.11 [69], we compared the homologous domains with the sequences that had the highest similarity to identify the 13 PCGs, and annotated the 13 PCGs under the condition that they could be translated correctly. The recognition of two rRNAs followed the same method as above. To gain a more intuitive visualization of the mitogenomes, we drew the circular mitochondrial maps, available on the web (cloudtutu.com.cn, accessed on 23 February 2024), and beautified the circular mitochondrial maps by using Affinity Designer 2 [70]. In addition, we used an online program (https://tandem.bu.edu/trf/trf.html, accessed on 13 April 2024) to check the tandem repeats in the A-T rich region [71], and calculated the relative synonymous codon usage (RSCU) of the 13 PCGs by using PhyloSuite v.1.2.3 [72]. DnaSP V6 [73] was used to estimate the nucleotide diversity (*Pi*) of the 13 PCGs and two rRNAs using a sliding window analysis of 200 bp with a step size of 20 bp. The complete mitogenomes were also utilized to calculate the genetic distance of the species by using the Kimura 2-parameter model in MEGA v.11. Nucleotide compositional bias was assessed using the AT-skew ((A − T)/(A + T)) and GC-skew ((G − C)/(G + C)) [74], and AliGROOVE v.107 [75] was applied to detect sequence heterogeneity.

### 2.4. Dataset Selection and Phylogenetic Analyses

To reconstruct the phylogenetic relationships of Heptageniidae within Ephemeroptera, we used 95 complete or nearly complete mitogenomes that included 17 newly sequenced mitogenomes and 78 mitogenomes (Table S1) downloaded from the NCBI website after filtering out poor quality mitogenomes, such as those containing a high degree of degenerate bases [13,16,17,19,20,21,23,24,25,29,61,76,77,78,79,80]. Furthermore, among the 78 downloaded sequences, there were representatives drawn from 15 families within Ephemeroptera. The 13 PCGs were extracted and aligned by the MAFFT plugin via PhyloSuite v.1.2.3. In addition, the Gblocks plugin in PhyloSuite v.1.2.3 was used to delete regions that were potentially highly variable, the regions with frequent insertions or deletions, or regions that could not be accurately aligned. Finally, we applied the plugin in PhyloSuite v.1.2.3 to concatenate the 13 PCGs, and we used the dataset of 13 PCGs to reconstruct the phylogenetic tree.

During the process of concatenating the gene fragments, the outputted files (‘concatenation.phy’ and ‘partition.txt’) were employed for selecting the best-fitting evolutionary model by using the software package PartionFinder 2.2.1 [81] within the Python environment, and the results all suggested “GTR + I + G’’ as the best evolutionary model. The results are presented in Table S2. On the basis of Bayesian inference (BI) and maximum likelihood (ML) analyses, we rebuilt phylogenetic trees, abbreviated as BI and ML trees, respectively. Due to the fact that Siphluriscidae often occupies a basal position [58,82], we selected *Siphluriscus chinensis* HQ875717 and *Siphluriscus* sp. 1 JZ-2022 ON729391 as the outgroup.

The ML tree was rebuilt by IQ-TREE v.2 [83] with 1000 runs and the ML + rapid bootstrap (BS) algorithm under the best evolutionary model. BI tree was rebuilt by MrBayes 3.2 [84] with the partition schemes obtained from PartionFinder 2.2.1. Markov Chain Monte Carlo (MCMC) analysis was used to compute 10 million generations. Every 1000 generations, a sample was taken, and the first 25% of the data was discarded. FigTree v.1.4.4 [85] was used to visualize the trees, and the software Affinity Designer 2 [70] was employed to beautify the trees.

### 2.5. Divergence Time Estimation

There are few reported fossils among Ephemeroptera species. Five fossil calibration points selected from reports [86,87,88,89] and a fossil website (www.fossilworks.org, accessed on 15 April 2024) were used to estimate the divergence time of Heptageniidae on the basis of the phylogenetic trees. Kohli et al. [37] recently estimated the divergence time within Ephemeroptera, and their results showed that the Ephemeroptera began to diverge at 267.14 Mya, which was close to the Late Carboniferous or Early Permian, so we set this time as the root age. We selected the fossil of Siphlonuridae (159–160.6 Mya) as the first fossil calibration point [86]. The second fossil calibration point (15.00–20.00 Mya) was *Borinquena schawallfussi* of Atalophlebinae (Leptophlebiidae) [88]. The third (98.17–99.41 Mya) and fourth (41.30–47.80 Mya) fossil calibration points used belong to the recorded fossil of Vietnamellidae and *Ephemerella* (Ephemerellidae), respectively [87,89]. The final fossil calibration point (48.60–55.80 Mya) of *Neophemera* (Neoephemeridae) was chosen by the fossil website (https://paleobiodb.org, accessed on 15 April 2024).

We calculated the evolutionary substitution rate by utilizing the Baseml subpackage within PAML 4.8 [90], then the mcmctree subpackage was used to calculate the length of each branch with ‘usedata = 3’, ‘RootAge < 2.6714’, and the GTR model. We then used a computational strategy of a burn-in period of 1,000,000, a sample frequency of 1000, and the number of samples being 10,000. To ensure the adequate operation of the MCMC chain, we used Tracer v.1.7.1 [91] to check whether the values of the effective sample size (ESS) were all greater than 200. The final divergence time of each branch was visualized in FigTree v.1.4.4 [85] and beautified using Affinity Designer 2 [85].

## 3. Results

### 3.1. Characteristics of Mitogenomes

**Figure 1:**
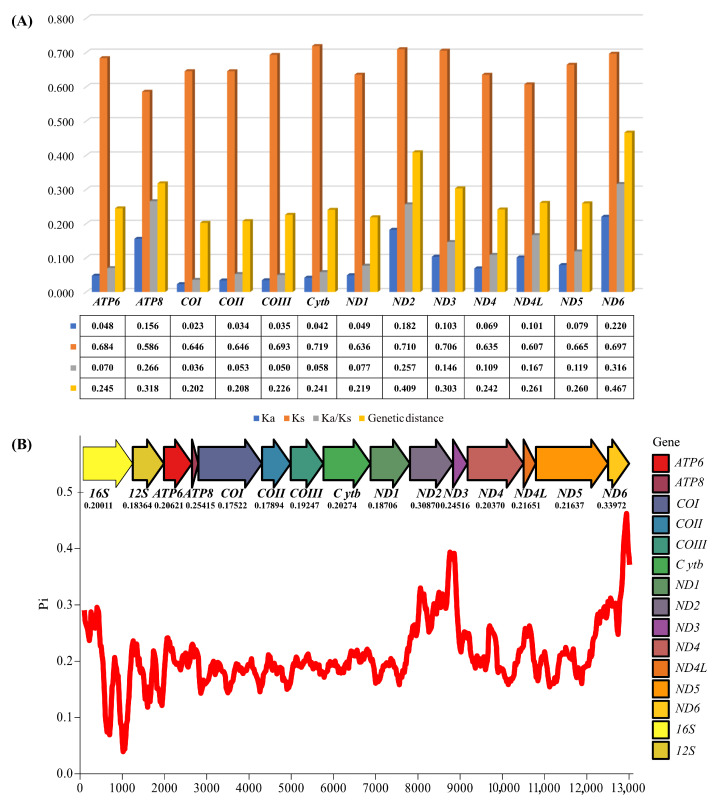
(**A**) The value of *Ka*, *Ks*, *Ka/Ks*, and the genetic distance of each gene. The different colored boxes represent different concepts, and the table contains specific values. (**B**) The value of *Pi* of each gene.

**Figure 2:**
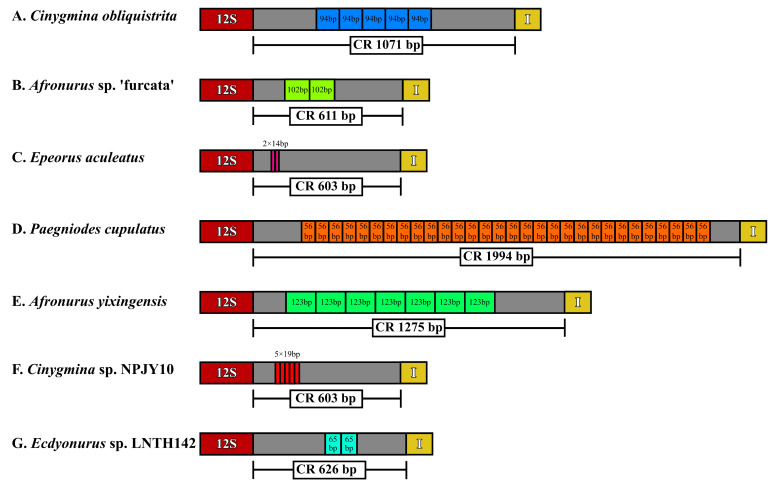
Schematic diagram of tandem repeat arrangements in the CR of seven mayfly species (**A**–**G**). And different colored boxes represent different genes.

The length of the 17 mitogenomes ranged from 15,249 bp for *Cinygmina obliquistrita* to 16,784 bp for *Pae. cupulatus*. The main reason for the difference in the lengths was the various lengths of the CR, which ranged from 405 bp in *C. obliquistrita* to 1994 bp in *Pae. cupulatus*. All mitogenomes encoded 13 PCGs, two rRNAs, and one CR, but due to the rearrangement of *trnI-trnM-trnQ-trnM*, except for *Pae. cupulatus* and *R. germanica*, which encoded 22 tRNAs, the remaining 15 mitogenomes all encoded 23 tRNAs. Among the 38 or 37 genes, 25 or 24 genes were encoded on the heavy strand (positive strand) and 13 genes were encoded on the light strand (negative strand). The RSCU of 15 mitogenomes (except two of *E. montanus*) is shown in Figure S2, and the results suggested that UUA (L), AUU (I), and UUU (F) had the highest usage. In this study, non-canonical start codons GTG appeared in *ND5*, which seemed to be an abnormal phenomenon, but also appeared in the vast majority of Heptageniidae species, such as the species of *Epeorus*. Furthermore, there were many different start codons of PCGs in the 17 mitogenomes, e.g., GTG in *ATP8* of *Afronurus yixingensis*, ACC in *COI* of *Afronurus* sp. ‘furcata’, TAC in *ND6* of *Parafronurus* sp. 16bf10, and so on (Table S3). From Table S3, we could see that incomplete codons (T and TA) also appeared, and codons mainly ending in T appeared in the *COI*, *COII*, *ND4*, and *ND5* genes. Moreover, the 17 mitogenomes in this study had high AT contents, especially in the CR and the third position of the codon. The AT-skew of the 13 PCGs in all species showed a negative value on both the heavy and light strands, whereas the GC-skew of most species was negative on the heavy strand and positive the on light strand. After combining the 13 PCGs and two rRNAs from the 17 mitogenomes to calculate the value of *Pi*, *Ka*, *Ks*, *Ka*/*Ks*, and the genetic distance (shown in Figure 1), the results indicated that the *ND6* (*Pi* = 0.33972), *ND2* (*Pi* = 0.30870), and *ATP8* (*Pi* = 0.25451) genes had the highest nucleotide diversity. Similarly, the genetic distance (on average) pointed to the highest genetic distance for *ND6*, *ND2*, and *ATP8*. Through detecting the repeat regions in the AT-rich region of the 17 newly sequenced mitogenomes, the CR of 7 mitogenomes showed different numbers and lengths of repeat regions (Figure 2). The *C. obliquistrita* mitogenome had 5 × 94 bp repeats, the *Afronurus* sp. ‘furcata’ mitogenome had 2 × 102 bp repeats, the *E. aculeatus* contained 2 × 14 bp repeats, and *Pae. cupulatus* contained 30 × 56 bp repeats. Furthermore, *A. yixingensis* had 7 × 123 bp repeats, *Cinygmina* sp. NPJY10 showed 5 × 19 bp repeats, and *Ecdyonurus* sp. LNTH142 had 2 × 65 bp repeats.

### 3.2. Characteristics of the Mitogenomes of E. montanus

**Figure 3:**
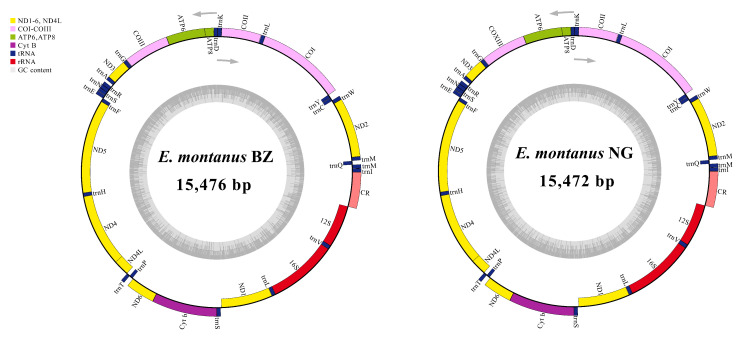
The circular mitochondrial maps of *E. montanus* BZ (**left**) and *E. montanus* NG (**right**). Genes located on the outermost circle are those found on the heavy strand, and those found on the inner circle are on the light strand.

**Figure 4:**
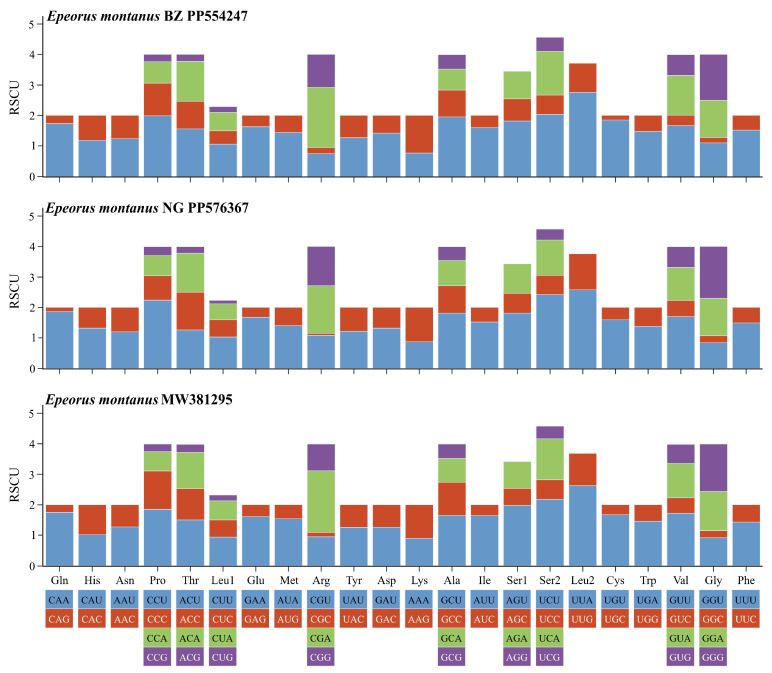
The RSCU of three mitogenomes of *E. montanus*.

Although three mitogenomes of *E. montanus* shared common features (such as each PCG sharing the same start codon and stop codon), slight differences still existed, such as the overall length of *E. montanus* BZ being longer than *E. montanus* MW381295, whereas the overall length of *E. montanus* NG was equal to *E. montanus* MW381295. Two complete mitogenomes of *E. montanus* BZ and *E. montanus* NG were collected from Aksu, Xinjiang Uygur Autonomous Region, and Xinyuan, Xinjiang Uygur Autonomous Region, respectively. The full mitogenome lengths of these were 15,476 bp and 15,472 bp, respectively. The circular mitochondrial maps of these two species are shown in Figure 3. To facilitate the comparison of mitogenomes in *E. montanus*, we compared the two mitogenomes obtained in this study with *E. montanus* MW381295. The three mitogenomes shared some features (Table S3), and features such as the AT content of three mitogenomes are shown in Table 2. Similar to other insect mitogenomes, the full mitogenomes of *E. montanus* BZ and *E. montanus* NG had a high AT content of 65.3% and 64.8%, respectively. *E. montanus* BZ had a base composition of 31.9% A, 33.4% T, 20.7% C, and 13.9% G. By contrast, the full mitogenome of *E. montanus* NG had a base composition of 31.6% A, 33.2% T, 21.3% C, and 13.9% G. In the two mitogenomes, both used the same start and stop codons, except for *ND5*, which used a special start codon (GTG), whereas all remaining PCGs used the standard start codons, ATG and ATT. For the stop codons, except for *COI*, *COII*, and *ND5*, which used the incomplete stop codon T as the stop codon, all other PCGs used complete stop codons: TAA or TCG. The results of RSCU (Figure 4) also showed that the UUA (L), AUU (I), and UUU (F) codons in the three mitogenomes had the most usage, all more than 200 times.

After we had calculated the genetic distance among some species (*E. herklotsi*, *E. aculeatus*, *Pae. cupulatus*, *C. obliquistrita*, *A. yixingensis*, *E. montanus*, and *Maccaffertium mediopunctatum*) in different communities, the results (Table S4) indicated that in other species (except *E. montanus*), the genetic distance between different communities did not reach the species level. However, we found that the genetic distance between the three mitogenomes of *E. montanus* ranged from 5.9% to 8.6%. The genetic distance of two species was 8.6%, and the genetic distance of *E. montanus* BZ and *E. montanus* NG from *E. montanus* MW381295 were 7.8% and 5.9%, respectively.

### 3.3. Phylogenetic Relationships of Heptageniidae

**Figure 5:**
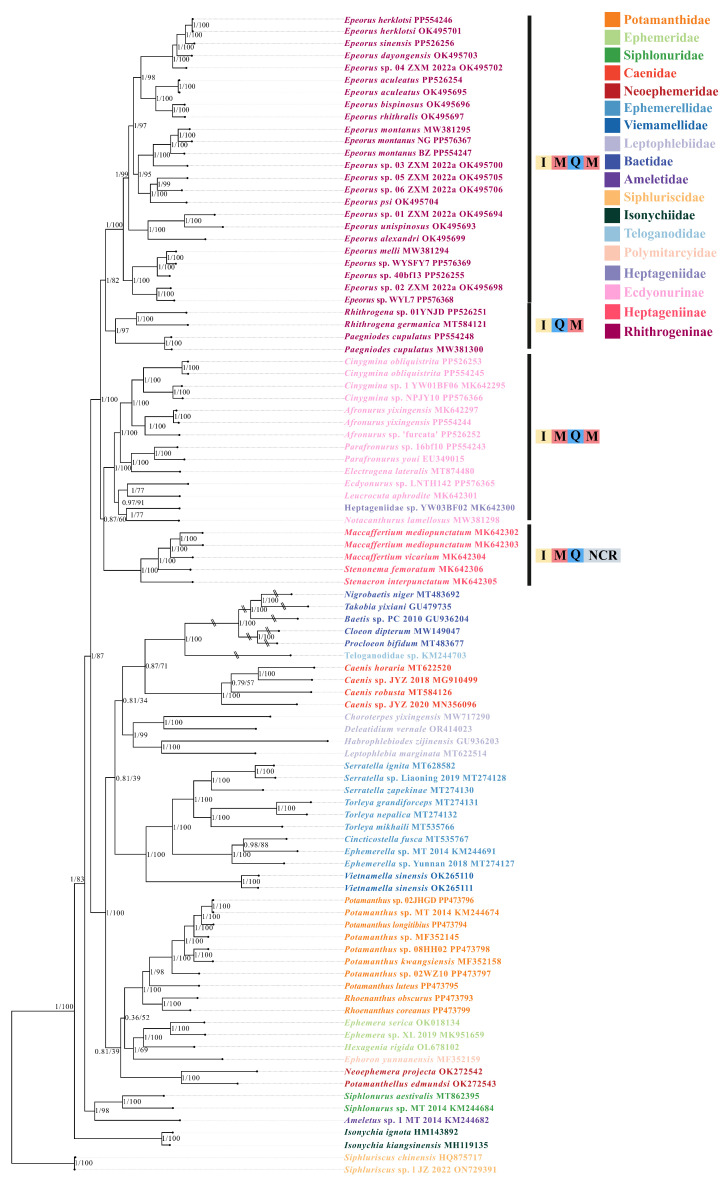
The results of phylogenetic analysis based on a tandem dataset of 13 PCGs of 95 mitogenomes (17 newly sequenced mitogenomes in this stfidy and 78 mitogenomes downloaded from NCBI). The posterior probability (**left**) and the bootstrap values (**right**) are shown on the nodes. Long branches in Baetidae and Teloganodidae were cut with double slash. The bars of I, Q, M, and NCR represent *trnI*, *trnQ*, *trnM*, and non-coding region, respectively.

In this study, 47 mitogenomes of Heptageniidae were used to explore the phylogenetic relationships among Heptageniidae. The topologies of BI tree and ML tree showed consistency (Figure 5). On the whole, two trees recovered the monophyly of all families except Polymitarcyidae and Ameletidae because of the utilization of a single mitogenome per family in this study. Long-branch attraction (LBA) in Baetidae and Teloganodidae was still found in two trees. After detecting sequence heterogeneity (Figure S3), we found that the sequences belonging to Baetidae and Teloganodidae had high heterogeneity, which may also be the reason for the occurrence of LBA.

At the family level, we recovered the monophyly of Heptageniidae, and found that Heptageniidae formed a sister group containing other families except for Siphluriscidae, Isonychiidae, Siphlonuridae, and Ameletidae. Isonychiidae formed a sister clade with an additional 13 families except for Siphluriscidae. Siphlonuridae and Ameletidae formed a sister group relationship and are hypothesized to have diverged the earliest from Isonychiidae. The clade of Potamanthidae + (Polymitarcyidae + Ephemeridae) and Neoephemeridae clustered to a sister clade, and the branch of (Potamanthidae + (Polymitarcyidae + Ephemeridae)) + Neoephemeridae was also supported as a sister clade to (((Baetidae + Teloganodidae) + Caenidae) + Leptophlebiidae) + (Ephemerellidae + Vietnamellidae). Within Heptageniidae, at the subfamily level, two trees recovered the monophyly of Ecdyonurinae, Heptageniinae, and Rhithrogeninae. In addition, two trees suggested the relationship of (Ecdyonurinae + Heptageniinae) + Rhithrogeninae with a high prior probability and bootstrap values. At the genus level, we recovered the monophyly of *Epeorus*, *Afronurus*, *Paegniodes*, *Rhithrogena*, *Cinygmina*, *Parafronurus*, and *Maccaffertium*. However, due to the limited number of mitogenomes within *Electrogena*, *Notacanthurus*, and *Leucrocuta*, the present study could not accurately resolve the monophyly of these genera. At the species level, two trees suggested the relationship of (*E. montanus* NG + *E. montanus* MW381295) + *E. montanus* BZ.

It is worth noting that the rearrangement of the three subfamilies had its own characteristics. Species of Heptageninae presented a rearrangement of *trnI-trnM-trnQ-NCR*, whereas Eedyonurinae presented a rearrangement of *trnI-trnM-trnQ-trnM*. Rhithrogeninae was a more specialized subfamily, with *Pae. cupulatus* and *R. germanica* having an ancestral gene arrangement. Remarkably, the two species came together as sister clades. Except for these two species, the remaining species of Rhithrogeninae had a rearrangement of *trnI-trnM-trnQ-trnM*.

### 3.4. Estimation of Divergence Time 

**Figure 6:**
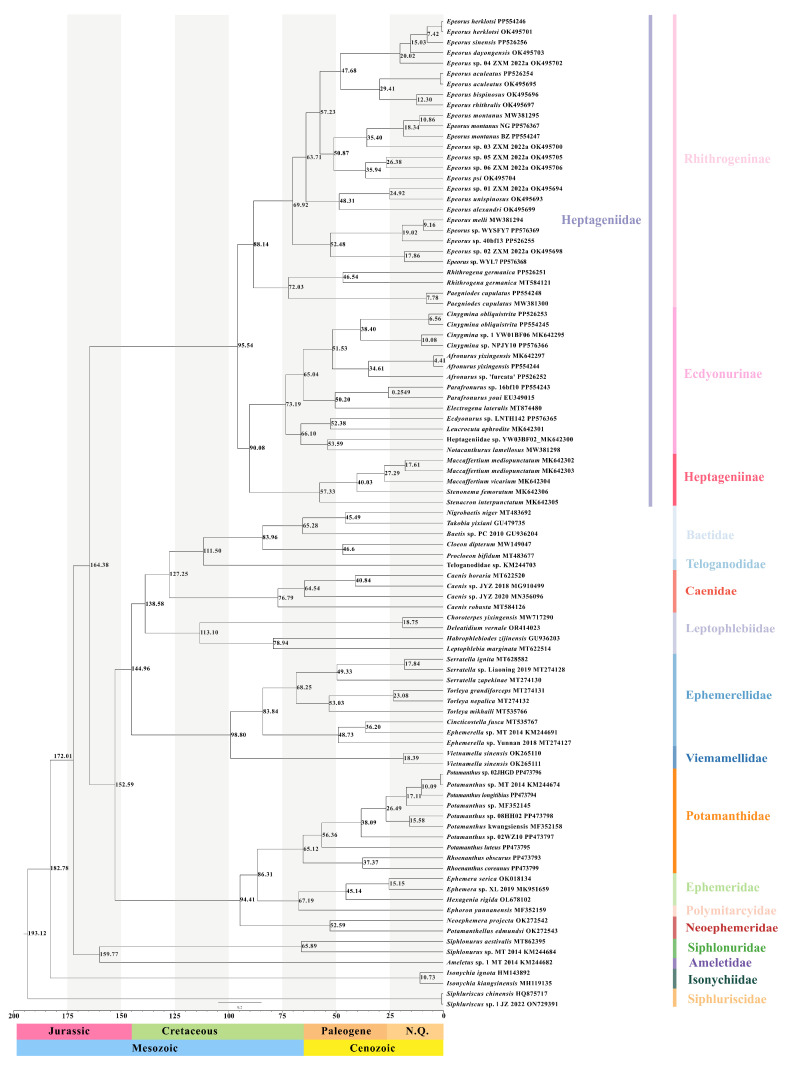
The results of divergence times based on the topology of ML and BI trees. The numbers above the nodes show the median ages.

We used the topology of the ML/BI tree based on 13 PCGs to analyze the divergence time. The results (Figure 6) showed that most families appeared in the Cretaceous (about 66–145 Mya), including Potamanthidae, Ephemeridae, Polymitarcyidae, Neoephemeridae, Ephemerellidae, Vietnamellidae, Baetidae, Teloganodidae, and Caenidae. Meanwhile Siphluriscidae, Isonychiidae, Heptageniidae, Siphluriscidae, Ameletidae, and Leptophlebiidae appeared in the Jurassic (about 199.60–145.50 Mya). Siphluriscidae diverged first at around 193.12 Mya (95% HPD, 167.53–201.58 Mya), Isonychiidae diverged next after Siphluriscidae at 182.78 Mya (95% HPD, 168.94–226.41 Mya). The results showed that Heptageniidae diverged at 164.38 Mya (95% HPD, 150.23–181.53 Mya) in the middle Jurassic. Ephemeridae and Polymitarcyidae were the two most recent families that appeared about 67.19 Mya (95% HPD, 48.49–86.80 Mya). The divergence time of each family is presented in Table S5. At the subfamily level, Rhithrogeninae began to diverge at around 95.54 Mya (95% HPD, 73.86–120.19 Mya) in the middle Cretaceous, whereas Ecdyonurinae and Heptageniinae originated 90.08 Mya (95% HPD, 68.81–113.16 Mya) in the middle Cretaceous. At the genus level, we found that *Epeorus* diverged 88.14 Mya (95% HPD, 67.02–109.56 Mya) in the late Cretaceous, and *Cinygmina* and *Afronurus* originated 51.53 Mya (95% HPD, 34.38–68.29 Mya). At the species level, we found that *E. montanus* BZ first diverged at 18.34 Mya (95% HPD, 9.96–28.71 Mya), then *E. montanus* NG + *E. montanus* MW381295 appeared at 10.86 Mya (95% HPD, 4.64–18.43 Mya).

## 4. Discussion

### 4.1. Phylogenetic and Gene Rearrangement Analyses

The use of mitogenomes to explore the phylogenetic relationships among higher taxonomic classifications is a well-established technique [13,14,15,18,19,20,21,22,23,24,25,58,61]. Although the phylogenetic relationships of Heptageniidae have been previously studied by multiple researchers, the current study is the first to reconstruct the phylogenetic relationships from the maximum number of Heptageniidae. Among these studies, Siphluriscidae was often used as an outgroup when constructing phylogenetic trees [14,16,20,21,79]. As in most studies, we recovered the monophyly of Heptageniidae in two phylogenetic trees with high bootstrap values and prior probabilities [13,14,15,16,18,19,20,21,79]. Heptageniidae was the sister clade to other families except for Siphluriscidae, Isonychiidae, Siphlonuridae, and Ameletidae, which is consistent with many studies [18,19,20,22,23,24] but different from Cai et al. [13], Yu et al. [25], and Odgen et al. [26]. Furthermore, LBA also appeared in this study, which was once thought to be due to large differences in the AT content. However, after detecting sequence heterogeneity, we found that it may be due to the strong heterogeneity of sequences leading to LBA [54]. The monophyly of three subfamilies was recovered, and we also recovered the relationship of (Ecdyonurinae + Heptageniinae) + Rhithrogeninae, which was consistent with Webb et al. [31] and Wang et al. [32] at the subfamily level, but was different from the relationship of Heptageniinae + (Ecdyonurinae + Rhithrogeninae) that was supported by Xu et al. [21] and Tong et al. [19].

Various rearrangements of genes are often considered to be related to phylogenetic information [92,93]. All species in Ecdyonurinae and Heptageniinae had rearrangements of *trnI*-*trnM*-*trnQ*-*trnM* and *trnI*-*trnM*-*trnQ*-*NCR*, and a shared gene rearrangement of *trnI*-*trnM*-*trnQ* indicated that Ecdyonurinae and Heptageniinae had a close relationship. Except for the species of *Rhithrogena* and *Paegniodes*, which clustered into a sister clade, the remaining species all had the gene cluster of *trnI*-*trnM*-*trnQ*-*trnM*, which could always be explained by the “tandem duplication and randon loss model”. This means that tandem repeats appeared first in *trnI-trnQ-trnM*, and formed the *trnI-trnQ-trnM-trnI-trnQ-trnM* gene cluster that, after the random loss of two extra genes (*trnQ* and *trnI*), produced the final rearrangement of *trnI-trnM-trnQ-trnM*. This rearrangement mechanism is also present in some species of Heptageniidae, but the only difference is the latter *trnM* has a certain mutation to form the final *NCR*. On the basis of the facts above, we inferred that the ancestral gene cluster of the Heptageniidae is *trnI*-*trnM*-*trnQ*-*trnM*, whereas the branch of *Rhithrogena* and *Paegniodes* contains the oldest features. Since there are few mitogenomes available in the Heptageniinae, this study further expands the representative species of each subfamily and genus in Heptageniidae, which will contribute to a better understanding of the evolution, phylogeny, gene arrangement, and gene rearrangement of Heptageniidae.

### 4.2. The Evolutional Time of Ephemeroptera

High concentrations of CO_2_ in the Jurassic caused by the Permian mass extinction event has long been thought to be the main cause of the Jurassic period’s prominent warming [94]. At the same time, plants underwent important developments in the early Jurassic, and the presence of these plants provided more oxygen to the environment [95]. Furthermore, fresh water increased strongly during the late Jurassic, with about one-third to one-half of it stored on the continent (the rest as ice), which also provided more habitats for mayflies to live [96]. All of these provided favorable climatic conditions for the appearance of multiple families of Ephemeroptera in the Jurassic. With the ready availability of foods such as phytoplankton and diatoms that also appeared in the Jurassic and Cretaceous, as well as the abundant algae during the Cretaceous period [5,97,98], these changes could readily promote the appearance of multiple families among Ephemeroptera.

The results of this study show that most families of mayflies originated from the Cretaceous period, which is consistent with Sroka et al., who suggested that Ephemeroptera species had a high origination rate during the Cretaceous period [35]. Most extant mayflies are a good food source, providing protein, minerals, B vitamins, and essential amino acids, with a low fat content, and can always serve as food for dragonflies, stoneflies, and fish in the ecosystem [3]. With the aquatic insects of Odonata and Plecoptera, fish also appeared in large numbers during the Cretaceous period [30], and a hypothesis of an “arms race” between mayflies and Odonata (or Plecoptera, or fish) was put forward to explain the coevolution of mayflies in response to Odonata (or Plecoptera, or fish) predation. The radiation of these “food” species may have contributed to the development and radiation of mayflies via coevolution between predator and prey. Furthermore, the escape of mayflies during the coevolution of Ephemeroptera and Odonata (or Plecoptera, or fish) could have increased the diversity of Ephemeroptera. Similar phenomena have been observed in the speciation of European whitefish (*Coregonus lavaretus*), which were driven by large-gape predators (e.g., *Esox lucius*) [37,99], and inland snails (*Karaftohelix*), which underwent morphological and behavioral diversification caused by predation [100]. Moreover, the evolution and distribution of biodiversity in mountainous regions are intimately connected with the formation and geology of mountains, as in mayflies from the Caucasus Mountains [101,102] and Alpine mayflies [38]. The formation of the Tianshan Mountains, which are located in Xinjiang Uygur Autonomous Region, are always considered to be associated with plate subduction and collisional orogenesis during the Paleozoic and the far-field effects of the India–Asia collision in the Cenozoic [103]. In this study, the collection sites of *E. montanus* NG and *E. montanus* BZ were across the Tianshan Mountains (Xinyuan County is in the north of the Tianshan Mountains, and Aksu is in the south of the Tianshan Mountains). And the results of calculating the divergence time showed that the two species diversified during the Cenozoic, period in which the Tianshan Mountains were uplifted. In light of the points above, this study speculated that it was the strong mountain activity that led to the two species’ diversification.

### 4.3. Identification of Cryptic Species

Morphologically, we found that there were no morphological differences between *E. montanus* BZ and *E. montanus* NG. Although three mitogenomes of *E. montanus* shared some features, slight differences still existed, such as the overall length of *E. montanus* BZ, which was longer than *E. montanus* MW381295, whereas the overall length of *E. montanus* BZ was equal to that of *E. montanus* MW381295. In addition to the length of the CR, the composition of each base, and other minor differences, the 13 PCGs of three mitogenomes had the same start codon and stop codon, and even the same length. The genetic distances of these three mitogenomes were 8.6% (*E. montanus* BZ-*E. montanus* NG), 7.8% (*E. montanus* BZ-*E. montanus* MW381295), and 5.9% (*E. montanus* NG-*E. montanus* MW381295). At the molecular level, Tong et al. [19] calculated the genetic distance of three mitogenomes of *Vietnamella sinensis* that ranged from 5.8% to 14.9%, and Williams found that the genetic distance of *Baetis rhodani* samples [44], which were sampled from different geographic locations in different countries, ranged from 8% to 19%. The genetic distance analysis indicated that *E. montanus* NG and *E. montanus* MW381295 had a close relationship, and the phylogenetic tree presented a similar result. After combining the morphological features with the molecular data, we suggest that the two species collected in this study may be cryptic species of *E. montanus*. Williams et al. used the method of calculating the genetic distance to judge the putative presence of cryptic species within *Baetis rhodani* from different sites, as did Tong et al. [18,19,44]. Furthermore, from the results of estimating the divergence time, we found that *E. montanus* BZ appeared 18.34 Mya (95% HPD, 9.96–28.71 Mya) earlier than other two species, which originated 10.86 Mya (95% HPD, 4.64–18.43 Mya). According to the research methods we used in this study, we also speculated that cryptic species were widespread in Ephemeroptera, such as *M. mediopunctatum*. However, due to the failure to collect this species, we could not further identify it, and we will pay more attention to this species in future research. In addition, due to the long history of studies on the morphological description of *E. montanus* and the limitation of the amount of data in this study, we will further discuss and improve this study in subsequent research.

## 5. Conclusions

After combining the 17 mitogenomes with others released by the NCBI, we constructed two phylogenetic trees (the BI tree and ML tree) that shared the same topology. We recovered the monophyly of Heptageniidae. At the subfamily level, the monophyly of three subfamilies and the relationship of Rhithrogeninae + (Ecdyonurinae + Heptageniinae) were also recovered. On the basis of five fossil calibration points, we deduced a hypothesis that predator–prey relationships may have provided the evolutionary dynamics for the rapid differentiation in families of mayflies. Indeed, in light of the genetic distance, phylogenetic relationships, and divergence time, we suspect that two cryptic species of *E. montanus* exist.

## Figures and Tables

**Table 1:** Details of the 17 species used in this study.

Number	Species	Subfamily	Length	Sampling Localities	Accession No.
02WZ07	*Afronurus* sp. ‘furcata’	Ecdyonurinae	15,436	Taishun, Zhejiang	PP526252
FZZX10	*Afronurus yixingensis*	Ecdyonurinae	16,120	Fuzhou, Jiangxi	PP554244
02JZWC	*Cinygmina obliquistrita*	Ecdyonurinae	15,249	Zhuji, Zhejiang	PP554245
02WZ03	*Cinygmina obliquistrita*	Ecdyonurinae	15,916	Taishun, Zhejiang	PP526253
NPJY10	*Cinygmina* sp. NPJY10	Ecdyonurinae	15,447	Nanping, Fujian	PP576366
16bf10	*Parafronurus* sp. 16bf10	Ecdyonurinae	15,527	Longquan, Zhejiang	PP554243
LNTH142	*Ecdyonurus* sp. LNTH142	Ecdyonurinae	15,453	Tonghua, Liaoning	PP576365
07BF95	*Epeorus aculeatus*	Rhithrogeninae	15,466	Tonglu, Zhejiang	PP526254
02WYNT	*Epeorus herklotsi*	Rhithrogeninae	15,498	Lishui, Zhejiang	PP554246
40bf13	*Epeorus* sp. 40bf13	Rhithrogeninae	15,508	Wuyi, Zhejiang	PP526255
BZD4	*Epeorus montanus* BZ	Rhithrogeninae	15,476	Akesu, Xinjiang	PP554247
NLTYT6	*Epeorus montanus* NG	Rhithrogeninae	15,472	Xinyuan, Xinjiang	PP576367
02WZ01	*Epeorus sinensis*	Rhithrogeninae	15,508	Taishun, Zhejiang	PP526256
WYL7	*Epeorus* sp. WYL7	Rhithrogeninae	15,546	Taishun, Zhejiang	PP576368
WYSFY7	*Epeorus* sp. WYSFY7	Rhithrogeninae	15,496	Taishun, Zhejiang	PP576369
34bf05	*Paegniodes cupulatus*	Rhithrogeninae	16,784	Kaihua, Zhejiang	PP554248
01YNJD	*Rhithrogena germanica*	Rhithrogeninae	15,398	Jingdong, Yunnan	PP526251

**Table 2:** Mitogenomic features of *E. montanus*.

		*E. montanus* BZ PP554247				*E. montanus* NG PP576367				*E. montanus* MW381295			
Region	Strand	Length (bp)	AT%	AT-skew	CG-skew	Length (bp)	AT%	AT-skew	CG-skew	Length (bp)	AT%	AT-skew	CG-skew
Full mitogenome	Heavy	15,476	65.3	−0.023	−0.197	15,472	64.8	−0.025	−0.21	15472	64.8	−0.019	−0.21
PCG	Heavy	6882	62.9	−0.195	−0.167	6882	61.9	−0.205	−0.176	6882	61.9	−0.192	−0.186
	Light	4326	66.2	−0.227	0.279	4326	66.1	−0.226	0.296	4326	66	−0.225	0.27
tRNA	Heavy	987	64.8	−0.037	0.026	987	64.6	−0.022	0.009	986	64.7	−0.019	0.105
	Light	529	65.4	−0.012	0.279	529	65.8	−0.011	0.282	529	64.8	−0.009	0.28
rRNA	Light	2066	67.2	0.03	0.26	2065	67.1	0.029	0.261	2065	67.3	0.026	0.262

## Data Availability

Data to support this study are available from the National Center for Biotechnology Information (https://www.ncbi.nlm.nih.gov) (accessed on 20 May 2024). The GenBank numbers are PP526251–PP526256, PP554243–PP554248, and PP576365–PP576369.

## Supplementary Materials

The following supporting information can be downloaded at https://www.mdpi.com/article/10.3390/insects15100745/s1. Figure S1. Collection grounds for the various species, with rectangles of different shapes and colors representing different species. And the map information was downloaded from the website of the Ministry of Civil Affairs of the People’s Republic of China (https://www.mca.gov.cn/, accessed on 20 April 2024). Figure S2. The RSCU of the remaining 15 mitogenomes. Figure S3. Analysis of heterogeneity in the 95 Ephemeroptera species, based on two datasets. Table S1. Information about the samples used in this study and their NCBI GenBank accession numbers. Table S2. Partition schemes and the best evolutionary models obtained by PartionFinder 2.2.1. Table S3. Location of the features of all mitogenomes plus *E. montanus* MW381295. Table S4. Genetic distances of the 17 mitogenomes that we obtained, plus *E. montanus* MW381295 and some species that belonged to different communities, released by NCBI. Table S5. The divergence time of each family. Refs. [104,105] are cited in Supplementary Materials file
