# Supplementary material for: Mitogenome-Based Phylogeny with Divergence Time Estimates Revealed the Presence of Cryptic Species within Heptageniidae (Insecta, Ephemeroptera)
Source: Insects. 2024 Sep 26;15(10):745. doi: 10.3390/insects15100745 (PMC11509038; doi:10.3390/insects15100745)
Supplement: Supplementary file 1 [file insects-15-00745-s001.zip › Figure S3.pdf]

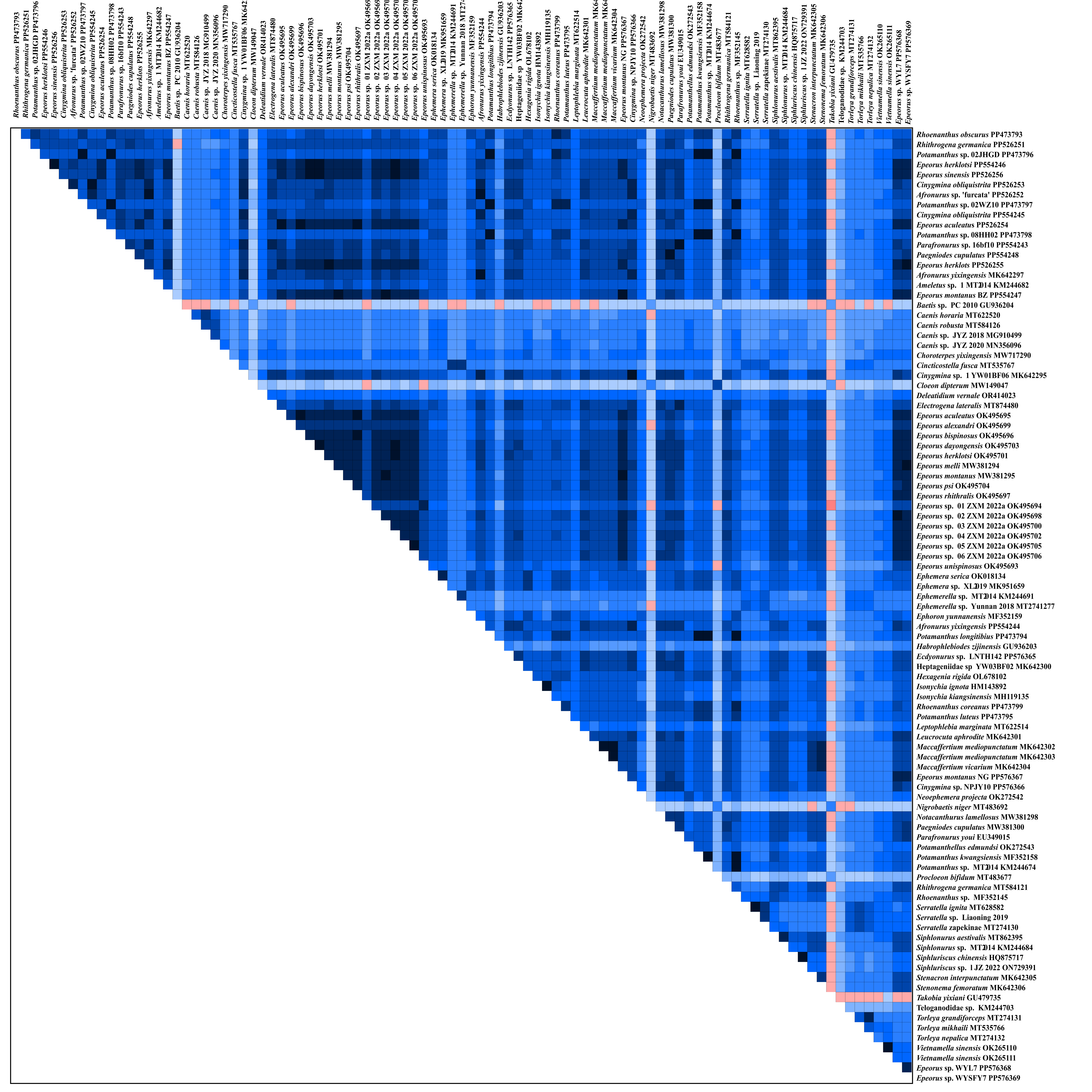

Rhoenanthus obscurus PP473793
Rhithrogena germanica PP526251
Potamanthus sp. 02JHGD PP473796
Epeorus herklotsi PP554246
Epeorus sinensis PP526256
Cinygmina obliquistrita PP526253
Afronurus sp. 'furcata' PP526252
Potamanthus sp. 02WZ10 PP473797
Cinygmina obliquistrita PP554245
Epeorus aculeatus PP526254
Potamanthus sp. 08HH02 PP473798
Parafronurus sp. 16bf10 PP554243
Paegniodes cupulatus PP554248
Epeorus herklots PP526255
Afronurus yixingensis MK642297
Ameletus sp. 1 MT2014 KM244682
Epeorus montanus BZ PP554247
Baetis sp. PC 2010 GU936204
Caenis horaria MT622520
Caenis robusta MT584126
Caenis sp. JYZ 2018 MG910499
Caenis sp. JYZ 2020 MN356096
Choroterpes yixingensis MW717290
Cincticostella fusca MT535767
Cinygmina sp. 1 YW01BF06 MK642295
Cloeon dipterum MW149047
Deleatidium vernale OR414023
Electrogena lateralis MT874480
Epeorus aculeatus OK495695
Epeorus alexandri OK495699
Epeorus bispinosus OK495696
Epeorus dayongensis OK495703
Epeorus herklotsi OK495701
Epeorus melli MW381294
Epeorus montanus MW381295
Epeorus psi OK495704
Epeorus rhithralis OK495697
Epeorus sp. 01 ZXM 2022a OK495694
Epeorus sp. 02 ZXM 2022a OK495698
Epeorus sp. 03 ZXM 2022a OK495700
Epeorus sp. 04 ZXM 2022a OK495702
Epeorus sp. 05 ZXM 2022a OK495705
Epeorus sp. 06 ZXM 2022a OK495706
Epeorus unispinosus OK495693
Ephemera serica OK018134
Ephemera sp. XL2019 MK951659
Ephemerella sp. MT2014 KM244691
Ephemerella sp. Yunnan 2018 MT2741277
Ephoron yunnanensis MF352159
Afronurus yixingensis PP554244
Potamanthus longitibius PP473794
Habrophlebiodes zijinensis GU936203
Ecdyonurus sp. LNTH142 PP576365
Heptageniidae sp YW03BF02 MK642300
Hexagenia rigida OL678102
Isonychia ignota HM143892
Isonychia kiangsinensis MH119135
Rhoenanthus coreanus PP473799
Potamanthus luteus PP473795
Leptophlebia marginata MT622514
Leucrocuta aphrodite MK642301
Maccaffertium mediopunctatum MK642302
Maccaffertium mediopunctatum MK642303
Maccaffertium vicarium MK642304
Epeorus montanus NG PP576367
Cinygmina sp. NPJY10 PP576366
Neoephemera projecta OK272542
Nigrobaetis niger MT483692
Notacanthurus lamellosus MW381298
Paegniodes cupulatus MW381300
Parafronurus youi EU349015
Potamanthellus edmundsi OK272543
Potamanthus kwangsiensis MF352158
Potamanthus sp. MT2014 KM244674
Procloeon bifidum MT483677
Rhithrogena germanica MT584121
Rhoenanthus sp. MF352145
Serratella ignita MT628582
Serratella sp. Liaoning 2019
Serratella zapekinae MT274130
Siphlonurus aestivalis MT862395
Siphlonurus sp. MT2014 KM244684
Siphluriscus chinensis HQ875717
Siphluriscus sp. 1 JZ 2022 ON729391
Stenacron interpunctatum MK642305
Stenonema femoratum MK642306
Takobia yixiani GU479735
Teloganodidae sp. KM244703
Torleya grandiforceps MT274131
Torleya mikhaili MT535766
Torleya nepalica MT274132
Vietnamella sinensis OK265110
Vietnamella sinensis OK265111
Epeorus sp. WYL7 PP576368
Epeorus sp. WYSFY7 PP576369
