# Supplementary material for: Mitogenome-Based Phylogeny with Divergence Time Estimates Revealed the Presence of Cryptic Species within Heptageniidae (Insecta, Ephemeroptera)
Source: Insects. 2024 Sep 26;15(10):745. doi: 10.3390/insects15100745 (PMC11509038; doi:10.3390/insects15100745)
Supplement: Supplementary file 1 [file insects-15-00745-s001.zip › Table S1.pdf]

**Table S1.** Information about samples used in this study and their NCBI GenBank accession numbers.

| S/N | Species | Family | Accession No. | Reference |
|---|---|---|---|---|
| 1 | *Ameletus* sp. 1 MT-2014 | Ameletidae | KM244682 | [76] |
| 2 | *Cloeon* dipterum | Baetidae | MW149047 | [77] |
| 3 | *Baetis* sp. PC-2010 | Baetidae | GU936204 | [78] |
| 4 | *Takobia yixiani* | Baetidae | GU479735 | [78] |
| 5 | *Nigrobaetis niger* | Baetidae | MT483692 | Direct Submission |
| 6 | *Procloeon bifidum* | Baetidae | MT483677 | Direct Submission |
| 7 | *Caenis* sp. JYZ-2020 | Caenidae | MN356096 | [23] |
| 8 | *Caenis* sp. JYZ-2018 | Caenidae | MG910499 | [13] |
| 9 | *Caenis horaria* | Caenidae | MT622520 | Direct Submission |
| 10 | *Caenis robusta* | Caenidae | MT584126 | Direct Submission |
| 11 | *Torleya mikali* | Ephemerellidae | MT535766 | [17] |
| 12 | *Cincticostella fusca* | Ephemerellidae | MT535767 | [17] |
| 13 | *Torleya nepalica* | Ephemerellidae | MT274132 | [25] |
| 14 | *Torleya grandifrceps* | Ephemerellidae | MT274131 | [25] |
| 15 | *Serratella zapekinae* | Ephemerellidae | MT274130 | [25] |
| 16 | *Ephemerella* sp. Yunnan-2018 | Ephemerellidae | MT274127 | [25] |
| 17 | *Serratella* sp. Liaoning-2019 | Ephemerellidae | MT274128 | [25] |
| 18 | *Serratella ignita* | Ephemerellidae | MT628582 | Direct Submission |
| 19 | *Ephemerella* sp. MT-2014 | Ephemerellidae | KM244691 | [76] |
| 20 | *Vietnamella sinensis* | Viemamellidae | OK265110 | [19] |
| 21 | *Vietnamella sinensis* | Viemamellidae | OK265111 | [19] |
| 22 | *Ephemera serica* | Ephemeridae | OK018134 | Direct Submission |
| 23 | *Hexagenia rigida* | Ephemeridae | OL678102 | [20] |
| 24 | *Ephemera* sp. XL-2019 | Ephemeridae | MK951659 | [103] |
| 25 | Heptageniidae sp. YW03BF02 | Heptageniidae | MK642300 | [21] |
| 26 | *Epeorus* sp. 06 ZXM-2022a | Heptageniidae | OK495706 | [29] |
| 27 | *Epeorus* sp. 05 ZXM-2022a | Heptageniidae | OK495705 | [29] |
| 28 | *Epeorus psi* | Heptageniidae | OK495704 | [29] |
| 29 | *Epeorus dayongensis* | Heptageniidae | OK495703 | [29] |
| 30 | *Epeorus* sp. 04 ZXM-2022a | Heptageniidae | OK495702 | [29] |
| 31 | *Epeorus herklotsi* | Heptageniidae | OK495701 | [29] |
| 32 | *Epeorus* sp. 03 ZXM-2022a | Heptageniidae | OK495700 | [29] |
| 33 | *Epeorus alexandri* | Heptageniidae | OK495699 | [29] |
| 34 | *Epeorus* sp. 02 ZXM-2022a | Heptageniidae | OK495698 | [29] |
| 35 | *Epeorus rhithralis* | Heptageniidae | OK495697 | [29] |
| 36 | *Epeorus bispinosus* | Heptageniidae | OK495696 | [29] |
| 37 | *Epeorus aculeatus* | Heptageniidae | OK495695 | [29] |
| 38 | *Epeorus* sp. 01 ZXM-2022a | Heptageniidae | OK495694 | [29] |
| 39 | *Epeorus unispinosus* | Heptageniidae | OK495693 | [29] |
| 40 | *Epeorus melli* | Heptageniidae | MW381294 | [79] |
| 41 | *Epeorus montanus* | Heptageniidae | MW381295 | [17] |

| | | | | |
|---|---|---|---|---|
| 42 | *Maccaffertium vicarium* | Heptageniidae | MK642304 | [21] |
| 43 | *Afronurus yixingensis* | Heptageniidae | MK642297 | [21] |
| 44 | *Cinygmina* sp. 1 YW01BF06 | Heptageniidae | MK642295 | [21] |
| 45 | *Rhithrogena germanica* | Heptageniidae | MT584121 | Direct Submission |
| 46 | *Electrogena lateralis* | Heptageniidae | MT874480 | Direct Submission |
| 47 | *Notacanthurus lamellosus* | Heptageniidae | MW381298 | [17] |
| 48 | *Maccaffertium mediopunctatum* | Heptageniidae | MK642302 | [21] |
| 49 | *Maccaffertium mediopunctatum* | Heptageniidae | MK642303 | [21] |
| 50 | *Maccaffertium vicarium* | Heptageniidae | MK642304 | [21] |
| 51 | *Stenacron interpunctatum* | Heptageniidae | MK642305 | [21] |
| 52 | *Stenonema femoratum* | Heptageniidae | MK642306 | [21] |
| 53 | *Leucrocuta aphrodite* | Heptageniidae | MK642301 | [21] |
| 54 | *Parafronurus youi* | Heptageniidae | EU349015 | [61] |
| 55 | *Isonychia kiangsinensis* | Isonychiidae | MH119135 | [24] |
| 56 | *Isonychia ignota* | Isonychiidae | HM143892 | Unpublished |
| 57 | *Choroterpes yixingensis* | Leptophlebidae | MW717290 | [16] |
| 58 | *Deleatidium vernale* | Leptophlebidae | OR414023 | Direct Submission |
| 59 | *Leptophlebia marginata* | Leptophlebidae | MT622514 | Direct Submission |
| 60 | *Habrophlebiodes zjiensis* | Leptophlebidae | GU936203 | [78] |
| 61 | *Potamanthellus edmundsi* | Neoephemeridae | OK272543 | Direct Submission |
| 62 | *Neoephemera projecta* | Neoephemeridae | OK272542 | Direct Submission |
| 63 | *Ephoron yunnanensis* | Polymitarcyidae | MF352159 | [25] |
| 64 | *Potamanthus kwangsiensis* | Potamanthidae | MF352158 | [25] |
| 65 | *Potamanthus* sp. | Potamanthidae | MF352145 | [25] |
| 66 | *Potamanthus* sp. MT-2014 | Potamanthidae | KM244674 | [25] |
| 67 | *Rhoenanthus obscurus* | Potamanthidae | PP473793 | [80] |
| 68 | *Rhoenanthus coreanus* | Potamanthidae | PP473799 | [80] |
| 69 | *Potamanthus* sp. 02JHGD | Potamanthidae | PP473796 | [80] |
| 70 | *Potamanthus* sp. 02WZ10 | Potamanthidae | PP473797 | [80] |
| 71 | *Potamanthus* sp. 08HH02 | Potamanthidae | PP473798 | [80] |
| 72 | *Potamanthus longitibius* | Potamanthidae | PP473794 | [80] |
| 73 | *Potamanthus luteus* | Potamanthidae | PP473795 | [80] |
| 74 | *Siphlonurus aestivalis* | Siphlonuridae | MT862395 | Direct Submission |
| 75 | *Siphlonurus* sp. MT-2014 | Siphlonuridae | KM244684 | [76] |
| 76 | *Siphluriscus chinensis* | Siphluriscidae | HQ875717 | [58] |
| 77 | *Siphluriscus* sp. I JZ-2022 | Siphluriscidae | ON729391 | [20] |
| 78 | Teloganodidae sp. | Teloganodidae | KM244703 | [76] |
