# Supplementary material for: Mitogenome-Based Phylogeny with Divergence Time Estimates Revealed the Presence of Cryptic Species within Heptageniidae (Insecta, Ephemeroptera)
Source: Insects. 2024 Sep 26;15(10):745. doi: 10.3390/insects15100745 (PMC11509038; doi:10.3390/insects15100745)
Supplement: Supplementary file 1 [file insects-15-00745-s001.zip › Table S2.pdf]

**Table S2.** Partition schemes and best evolutionary models obtained from PartionFinder 2.2.1.

| Subset | Subset partitions | Best model |
|---|---|---|
| **Nucleotide sequence alignments (A dataset of 13 PCGs in tandem)** | | |
| **Subset** | **Subset partitions** | **Best model** |
| Partition 1 | COXI_codon1, Cyt b_codon1, COXIII_codon1, ATP6_codon1 | GTR+I+G |
| Partition 2 | COXI _codon2, Cyt b_codon2, COXII_codon2, COXIII_codon2, ATP6_codon2 | TVM+I+G |
| Partition 3 | ATP8_codon3, COXII_codon3, ATP6_codon3 | K81UF+I+G |
| Partition 4 | ND3_codon1, ND6_codon1, ATP8_codon1, ND2_codon1 | TVM+I+G |
| Partition 5 | ATP8_codon2, ND2_codon2, ND6_codon2, ND3_codon2 | GTR+I+G |
| Partition 6 | COXI_codon1 | GTR+I+G |
| Partition 7 | COXI_codon3 | TRN+I+G |
| Partition 8 | Cyt b_codon3, COXIII_codon3 | TRN+I+G |
| Partition 9 | ND4L_codon1, ND5_codon1, ND4_codon1, ND1_codon1 | GTR+I+G |
| Partition 10 | ND5_codon2, ND4_codon2, ND1_codon2, ND4L_codon2 | GTR+I+G |
| Partition 11 | ND1_codon3, ND4_codon3, ND5_codon3 | GTR+G |
| Partition 12 | ND2_codon3, ND6_codon3, ND3_codon3 | GTR+G |
| Partition 13 | ND4L_codon3 | TIM+I+G |
