# Supplementary material for: Mitogenome-Based Phylogeny with Divergence Time Estimates Revealed the Presence of Cryptic Species within Heptageniidae (Insecta, Ephemeroptera)
Source: Insects. 2024 Sep 26;15(10):745. doi: 10.3390/insects15100745 (PMC11509038; doi:10.3390/insects15100745)
Supplement: Supplementary file 1 [file insects-15-00745-s001.zip › Table S5.pdf]

Table S5. The divergence-time of each family.

Family	Mean divergence-time (Mya)	95% HPD Range (Mya)
Heptageniidae	164.38	150.23-181.53
Potamanthidae	86.31	65.33-106.52
Ephemeridae & Polymitarcyidae	67.19	48.49-86.80
Baetidae & Teloganodidae	111.50	90.47-131.42
Caenidae	127.25	107.88-147.29
Neoephemeridae	94.41	72.95-119.70
Ephemerellidae & Viemamellidae	98.80	98.17-99.41
Leptophlebiidae	138.58	118.74-157.01
Ameletidae & Siphlonuridae	159.77	159.00-160.59
Isonychiidae	182.78	167.53-201.58
Siphluriscidae	193.12	168.94-226.41
