# Supplementary figures and images for: Mitogenome-Based Phylogeny with Divergence Time Estimates Revealed the Presence of Cryptic Species within Heptageniidae (Insecta, Ephemeroptera)
Source: Insects. 2024 Sep 26;15(10):745. doi: 10.3390/insects15100745 (PMC11509038; doi:10.3390/insects15100745)

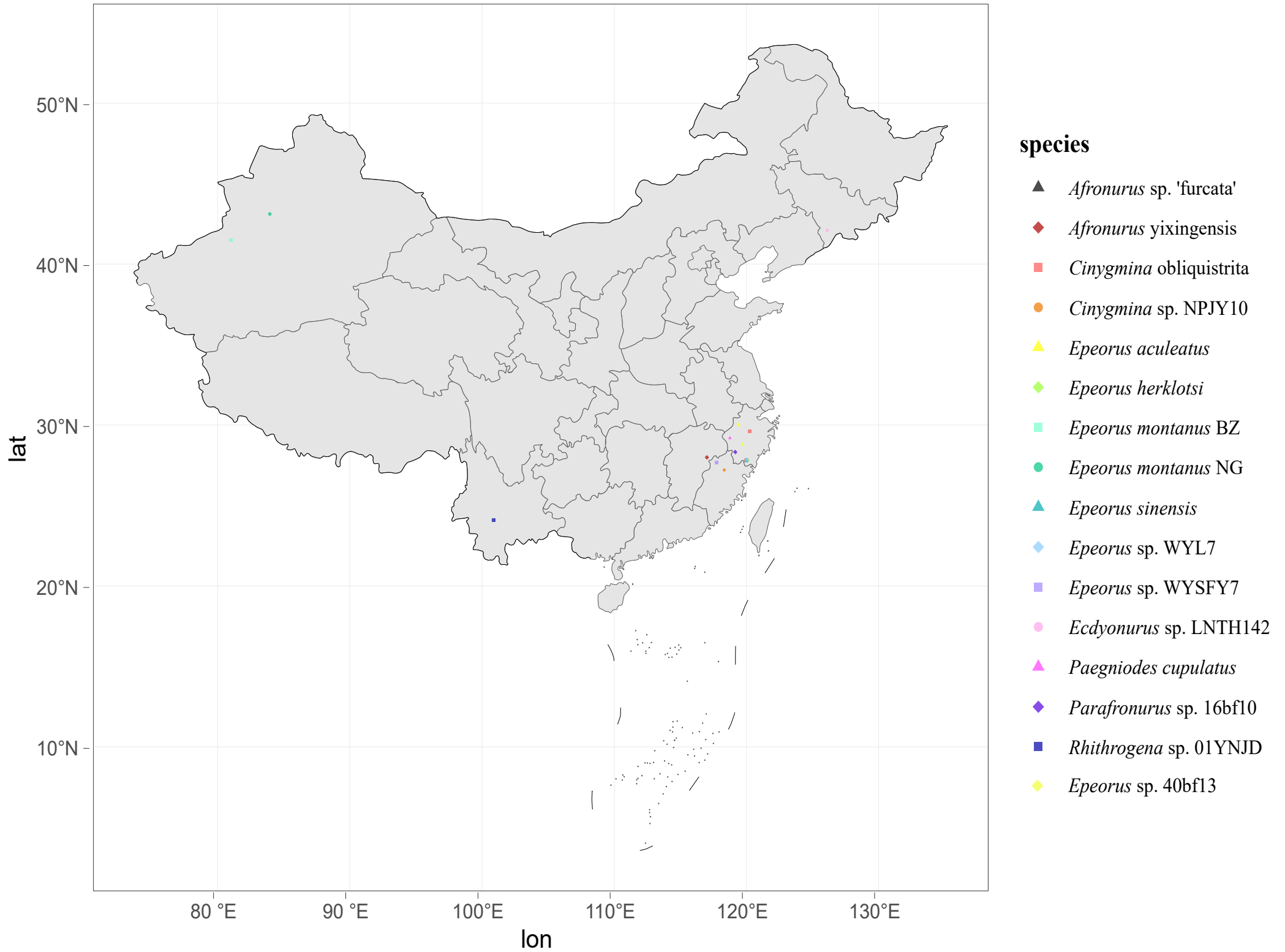

Supplement: Supplementary file 1 [file insects-15-00745-s001.zip › Figure S1.pdf]

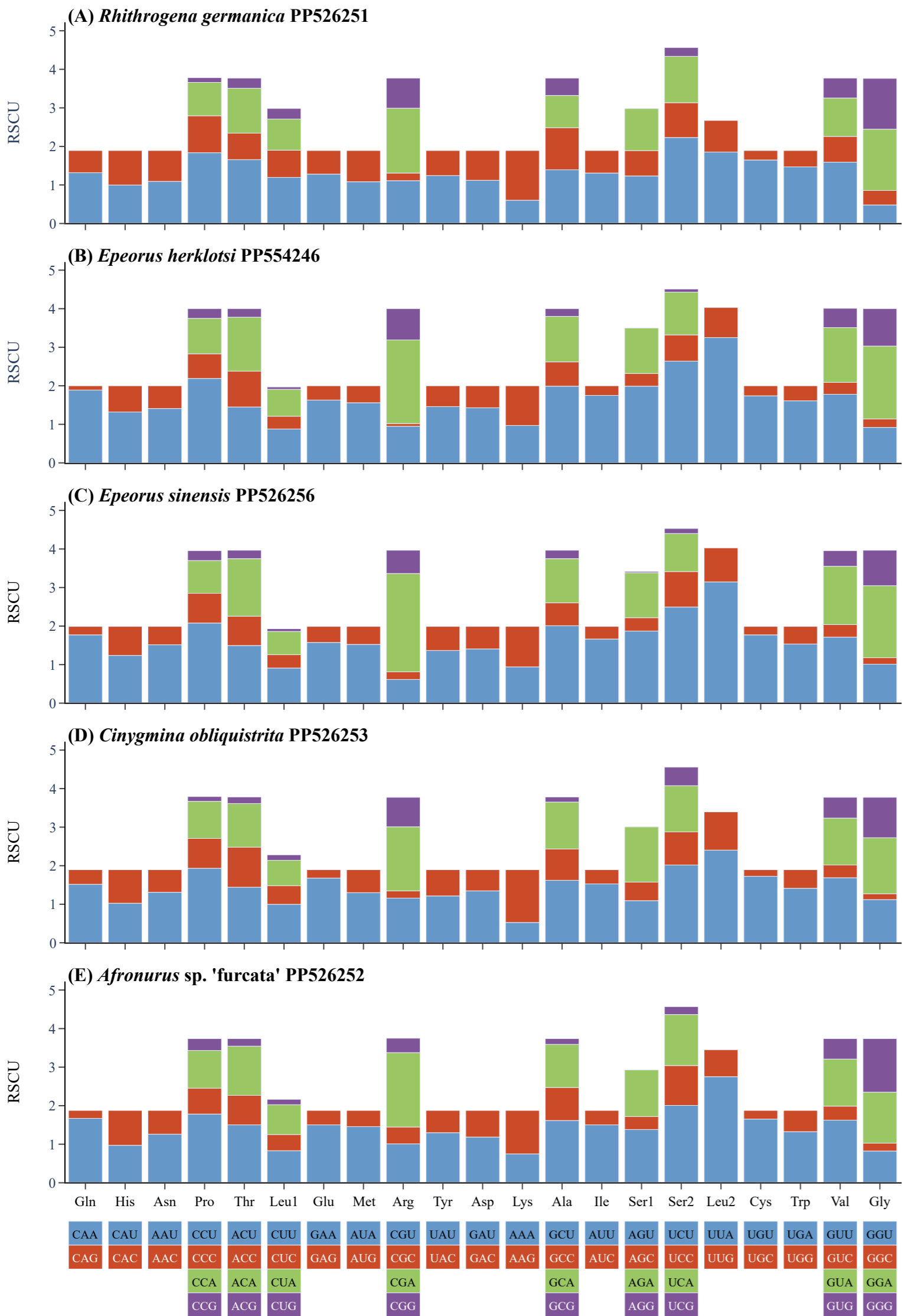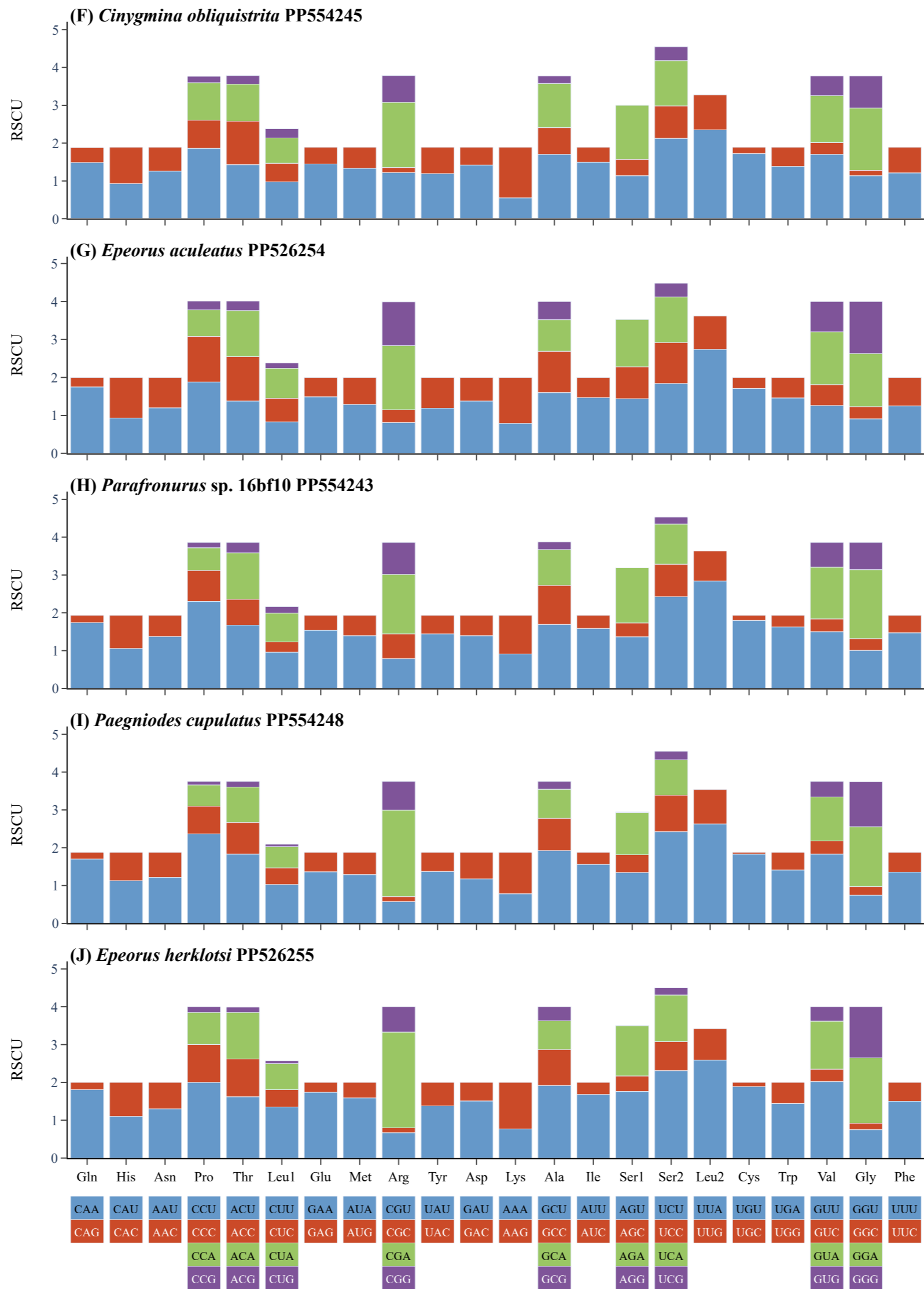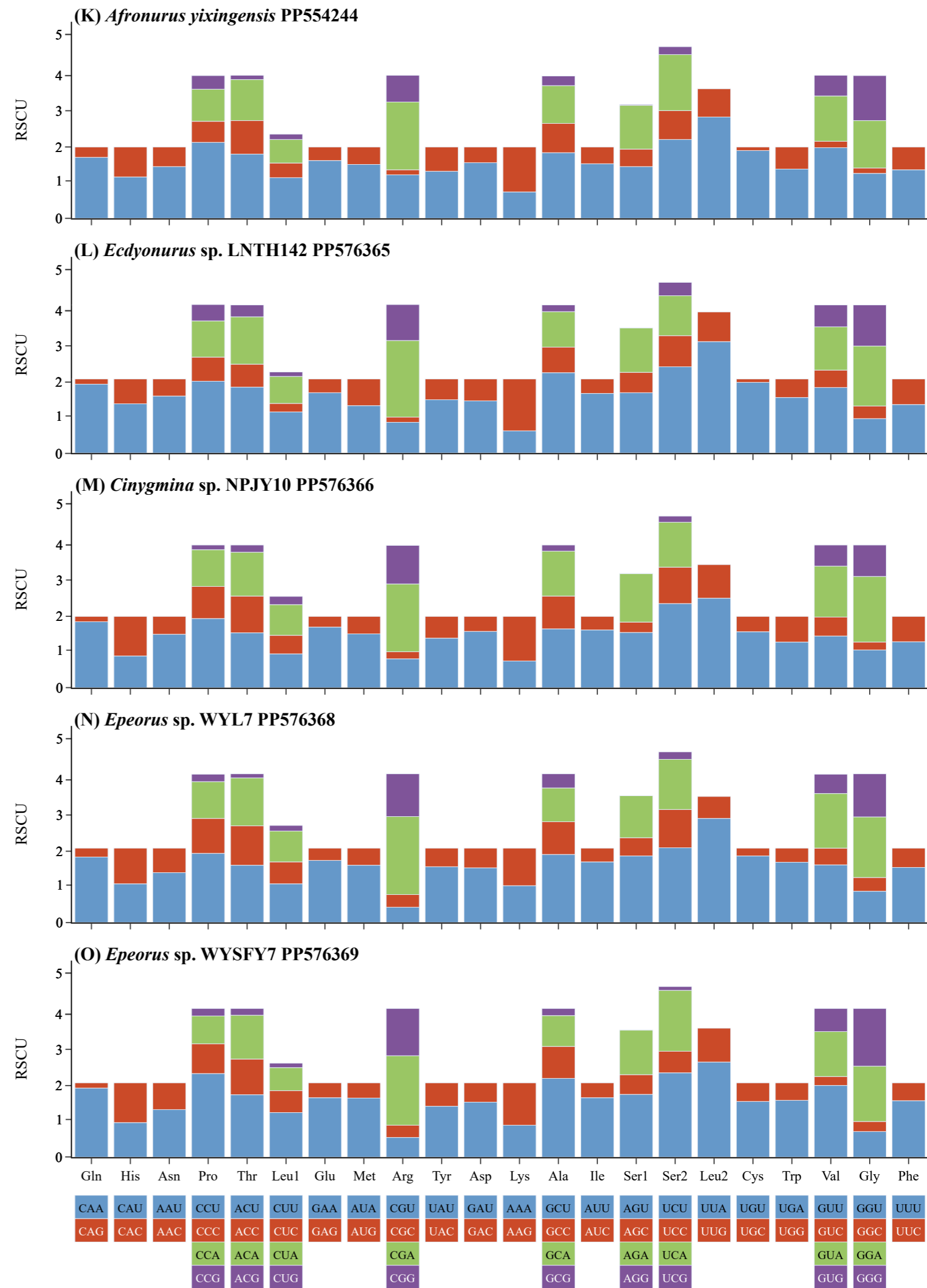

Supplement: Supplementary file 1 [file insects-15-00745-s001.zip › Figure S2.pdf]
